# The regulation of ferroptosis by MESH1 through the activation of the integrative stress response

**DOI:** 10.1038/s41419-021-04018-7

**Published:** 2021-07-22

**Authors:** Chao-Chieh Lin, Chien-Kuang Cornelia Ding, Tianai Sun, Jianli Wu, Kai-Yuan Chen, Pei Zhou, Jen-Tsan Chi

**Affiliations:** 1 Department of Molecular Genetics and Microbiology, Duke University Medical Center, Durham, NC USA; 2 Duke Center for Genomic and Computational Biology, Durham, NC USA; 3 Department of Biomedical Engineering, Duke University, Durham, NC USA; 4 Department of Biochemistry, Duke University School of Medicine, Durham, NC USA

**Keywords:** Enzyme mechanisms, Cell death, Transcription

## Abstract

All organisms exposed to metabolic and environmental stresses have developed various stress adaptive strategies to maintain homeostasis. The main bacterial stress survival mechanism is the stringent response triggered by the accumulation “alarmone” (p)ppGpp, whose level is regulated by RelA and SpoT. While metazoan genomes encode *MESH1* (Metazoan SpoT Homolog 1) with ppGpp hydrolase activity, neither ppGpp nor the stringent response is found in metazoa. The deletion of *Mesh1* in Drosophila triggers a transcriptional response reminiscent of the bacterial stringent response. However, the function of *MESH1* remains unknown until our recent discovery of *MESH1* as the first cytosolic NADPH phosphatase that regulates ferroptosis. To further understand whether *MESH1* knockdown triggers a similar transcriptional response in mammalian cells, here, we employed RNA-Seq to analyze the transcriptome response to *MESH1* knockdown in human cancer cells. We find that *MESH1* knockdown induced different genes involving endoplasmic reticulum (ER) stress, especially *ATF3*, one of the *ATF4*-regulated genes in the integrative stress responses (ISR). Furthermore, *MESH1* knockdown increased ATF4 protein, eIF2a phosphorylation, and induction of *ATF3*, *XBPs*, and *CHOP* mRNA. *ATF4* induction contributes to ~30% of the transcriptome induced by *MESH1* knockdown. Concurrent *ATF4* knockdown re-sensitizes *MESH1*-depleted RCC4 cells to ferroptosis, suggesting its role in the ferroptosis protection mediated by *MESH1* knockdown. *ATF3* induction is abolished by the concurrent knockdown of *NADK*, implicating a role of NADPH accumulation in the integrative stress response. Collectively, these results suggest that *MESH1* depletion triggers ER stress and ISR as a part of its overall transcriptome changes to enable stress survival of cancer cells. Therefore, the phenotypic similarity of stress tolerance caused by *MESH1* removal and NADPH accumulation is in part achieved by ISR to regulate ferroptosis.

## Introduction

Nutrient deprivation and various forms of environmental stresses impact the physiology and survival of organisms. To maintain homeostasis and survival under metabolic stresses, all organisms have developed adaptive strategies to cope with these adverse conditions. When bacteria are confronted with low nutrient availability, these metabolic stresses lead to the accumulation of guanosine 3′,5′-bidiphosphate (pppGpp or ppGpp), which triggers the ‘stringent response’ as a distinct metabolic state [1, 2]. (p)ppGpp binds to RNA polymerase and dramatically alters transcriptome to mediate a stress survival expression program. In addition, (p)ppGpp also inhibits translation initiation by binding to the IF2 (initiation factor 2) [3]. The levels of (p)ppGpp are regulated by its synthesis (by RelA) and degradation (by SpoT) [1, 2]. While the stringent response is highly conserved among bacteria and plants, no stringent response has been reported in mammals [4].

In multi-cellular organisms, the microenvironments of most cells are usually maintained within narrow physiological ranges. However, such “normal” physiological conditions can be disrupted by various disease processes, including vascular blockage during cardiac ischemia or stroke that would lead to a rapid depletion of oxygen, glucose as well as accumulation of lactic acids and other metabolic wastes. Similarly, the microenvironments of solid tumors may also exhibit glucose and amino acid deprivation, hypoxia, and lactic acidosis, which are caused by defective blood vessels, poor vascular perfusion, and overgrowth of the tumor cells. In mammalian cells, different stress conditions lead to distinct stress responses to coping with specific metabolic challenges. For example, under limiting oxygen, cells develop a hypoxia response triggered by hypoxia-inducible factors that mediate various metabolic changes to cope with the limited oxygen supply [5–7]. Similarly, lactic acidosis from tumor hypoxia and anaerobic glycolysis trigger metabolic reprogramming to limit glycolysis [8–11]. Glucose deprivation leads to energy depletion, increased AMP/ATP ratios, and subsequent *AMPK* activation that limits biosynthesis through the phosphorylation of acetyl-CoA carboxylase 1 (ACC1) [12]. Over time, these stress conditions also select tumors with genetic changes that offer survival advantages under stresses [10, 13]. Other than these stress-specific responses, mammalian cells also have a conserved and stereotypic stress response pathway named the endoplasmic reticulum (ER) stress or unfolding protein response (UPR). ER stress can be initiated by amino acid deprivation [14], hypoxia, and lactic acidosis [10] as well as other stresses [15]. ER stress or UPR are usually divided into three parallel and distinct branches as defined by signaling proteins in ER: PERK (PKR–like ER kinase), IRE1 (inositol requiring enzyme 1), and activating transcription factor 6 (ATF6). A PERK branch mediates an integrative stress response (ISR) [15], which is characterized by the phosphorylation of the eIF2α (alpha subunit of eukaryotic translation initiation factor 2) that reduces the global protein synthesis while allowing the preferential translation of *ATF4*. *ATF4* functions to induce the transcription of another transcription factor, *CHOP* (GADD153/DDIT3). Together, *ATF4* and *CHOP* enhance the transcriptional expression of genes involved in amino acid metabolism and resistance to oxidative stress.

Although the stringent response has not been reported in metazoa or mammals, the metazoan genome contains *MESH1* (Metazoan homologs of SpoT), a homolog of bacterial *SpoT* that possesses the enzymatic activities of the ppGpp hydrolase [16]. However, (p)ppGpp is barely detectable in metazoan cells. The genetic deletion of *Mesh1* in Drosophila shows upregulation of stress-responsible genes, suggesting the presence of a bacterial stringent-like stress response in metazoa [16]. Recently, we identified *MESH1* as the first cytosolic NADPH phosphatase [17] which was induced by erastin treatment or cystine deprivation [18]. *MESH1* knockdown robustly protected cells from ferroptosis, a novel form of iron-dependent cell death characterized by lipid peroxidation [18, 19]. Ferroptosis is triggered by a wide variety of environmental stresses experienced by mammalian cells. *MESH1* was identified by the functional genomic screens during cystine-deprived death [20]. Therefore, similar to the SpoT inhibition to protect bacterial cells from stress death, *MESH1* removal also protected human cells from ferroptosis induced by extreme oxidative stresses. While *MESH1* removal leads to stress tolerance phenotypes, much remains unknown about the transcriptional response of *MESH1* removal.

The transcriptional response is a prominent feature of a bacterial stringent response. Since the deletion of *Mesh1* in *Drosophila* led to transcriptional response reminiscent of bacterial stringent response [16] and the knockdown of *MESH1* conferred robust protection against ferroptosis [18], we wished to understand the transcriptional response to *MESH1* knockdown in human cells. Here we report that as part of the transcriptional response to *MESH1* knockdown, it triggers a prominent feature of the ER stress response with repression of cell proliferation and the ATF4 activation by ISR contributing to ferroptosis protection under *MESH1* knockdown. These findings suggest *MESH1* removal triggers a stress response that helps mammalian cells to cope with extreme oxidative stresses and prevents ferroptosis.

## Results

### *MESH1*-knockdown induces an extensive transcriptional response

**Fig. 1:**
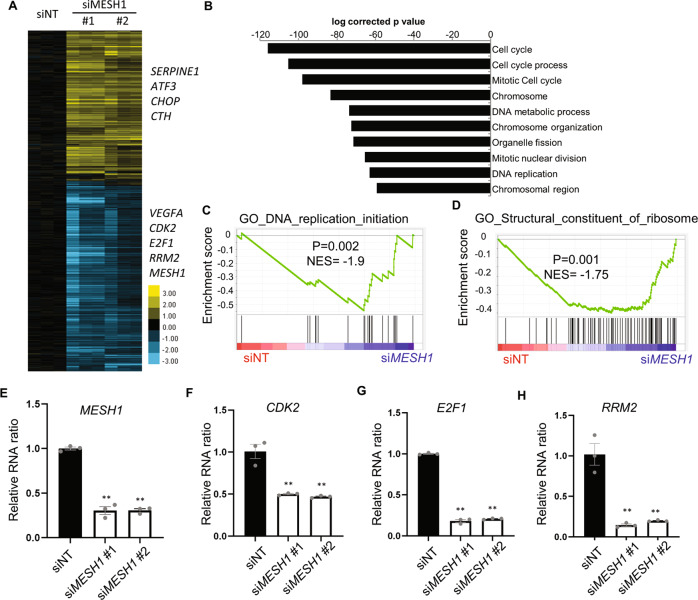
*MESH1* knockdown suppresses a transcriptional response of cell cycle progression. **A** Heatmap of the transcriptional difference between control or two independent MESH1 siRNA knockdown in RCC4 cells. *MESH1* was silenced by siMESH1#1 or siMESH1#2 and profiled by RNA-seq. Color scale indicates log2-fold-change. **B** Top ten repressed Gene Ontology processes of the transcriptome of *MESH1*-silenced RCC4 cells. **C**, **D** GSEA analysis showed the enrichment of DNA replication geneset (**C**) and constituent of ribosome (**D**) in the *MESH1* knockdown cells. **E**–**H** mRNA abundance of *MESH1* (**E**), *CDK2* (**F**), *E2F1* (**G**), and *RRM2* (**H**) as validated by rt-qPCR. mRNA abundance was determined by rt-qPCR, normalized by β-actin, and presented in relative ratio to siNT (non-targeting) treatment. *n* = 3 biological replicates. Statistical analysis: ANOVA with Tukey HSD post hoc test, **P < 0.01.

The bacterial stringent response is characterized by extensive transcriptome changes [1]. Similarly, the depletion of Drosophila *Mesh1* also triggered dramatic transcriptional changes reminiscent of bacterial stringent response [16]. Since the knockdown of human *MESH1* offered strong ferroptosis protection, we wished to investigate the transcriptional response to *MESH1* knockdown to (1) understand the ferroptosis protection phenotypes and (2) explore their potential similarity with these previously reported features of bacterial stringent response by removal of Drosophila *Mesh1*. We employed RNA-Seq to profile the transcriptome of RCC4 cells transfected with either control or two independent *MESH1* siRNAs that efficiently knocked down *MESH1* RNA expression (GEO: GSE114282). The successful knockdown of *MESH1* was confirmed in the RNA-Seq data (Fig. 1A). The transcriptome analysis revealed that *MESH1* knockdown triggered extensive transcriptional responses, including the downregulation of several genes in the DNA synthesis and cell cycle progression, including *CDK2* (Cyclin-dependent kinase 2), *E2F1* (E2F transcription factor 1), and *RRM2* (Ribonucleotide Reductase Regulatory Subunit M2) (Fig. 1A). Next, the Gene Ontology (GO) analysis confirmed that *MESH1* knockdown repressed pathways associated with cell cycle progression and DNA replication (Fig. 1B, Supplementary Tables 1 and 2). Gene set enrichment analysis (GSEA) revealed that *MESH1* knockdown repressed DNA replication, which was also one of the features of stringent response in *Escherichia coli* [21] (Fig. 1C, Supplementary Tables 1 and 2). In addition, *MESH1* knockdown also repressed several gene sets associated with ribosome structural constituents and organelles, implying reduced translation (Fig. 1D). To validate the results of RNA-seq, we knocked down *MESH1* by control or two independent *MESH1* siRNA in RCC4 cells (Fig. 1E) and used quantitative real-time polymerase chain reaction (qRT-PCR) to confirm the significant repression of cell cycle progression genes, including *CDK2* (Fig. 1F), *E2F1* (Fig. 1G), and *RRM2* (Fig. 1H). RRM2 encodes a subunit of ribonucleotide reductase which provides the dNTPs required for DNA synthesis. Interestingly, the bacterial stringent response in *E. coli* and *Mesh1*-deficient Drosophila also caused similar cell cycle arrest [16, 21–23]. These data suggest that *MESH1* knockdown in mammalian cells triggers a transcriptional response of cell cycle arrest and reduced ribosomal activities with a striking similarity to those observed in the bacterial stringent response [22, 23] and *Mesh1* deficient Drosophila [16]. Among the transcriptional response (Fig. 1A), we are particularly interested in the upregulation of *ATF3* (Fig. 1A), considering its role as a canonical target gene of the ISR, a PERK branch of the ER stress [15].

### *MESH1* knockdown triggers an ER stress response

**Fig. 2:**
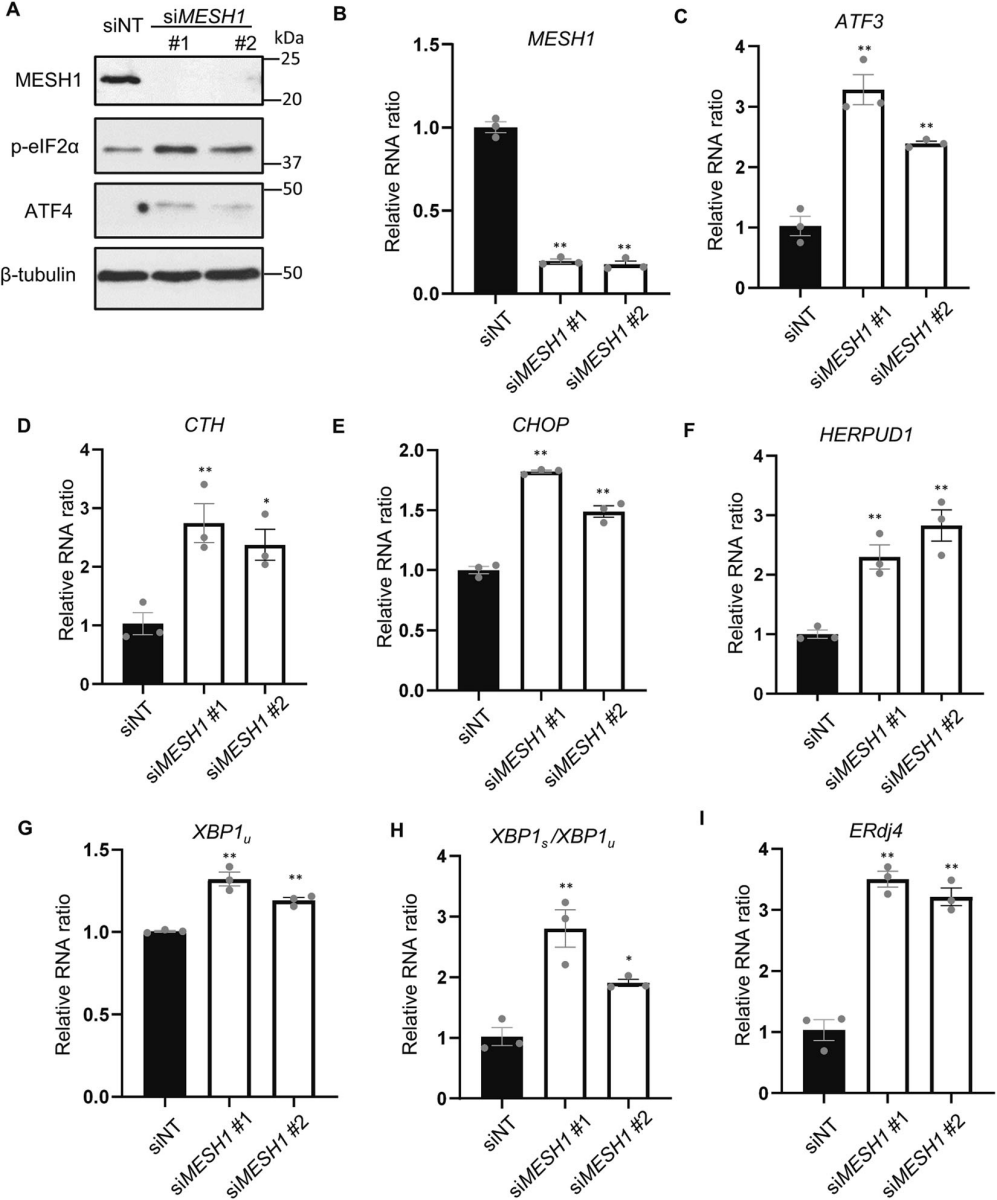
*MESH1* knockdown activates the ER stress response. **A** *MESH1* knockdown increased phosphorylated eIF2α protein and ATF4 protein expression. RCC4 cells knocked down by non-targeting (NT) siRNA or two independent *MESH1* siRNA for three days were lysed for WB. β-actin as a loading control. **B**–**I** *MESH1* knockdown (**B**) increased mRNA expression of several genes involving in the PERK pathway, including *ATF3* (**C**), *CTH* (**D**), *CHOP* (**E**), ATF6 target genes: *HERPUD1* (**F**), and total *XBP1* (*XBP1u*) (**G**), IRE1 target gene: spliced XBP1(*XBP1s*) as determined by *XBP1s*/*XBP1u* ratio (**H**) and ERdj4 (**I**). *n* = 3 biological replicates. Statistical analysis: ANOVA with Tukey HSD post hoc test, *P < 0.05, **P < 0.01.

The main function of the bacterial stringent response is to maintain survival and homeostasis under metabolic stresses and nutrient deprivations. One similar stress survival process in mammalian cells is ER stress [15] triggered by a wide variety of stress conditions. A PERK branch of ER stress, or ISR, is characterized by the eIF2α phosphorylation, ATF4 protein stabilization, transcriptional induction of *ATF3*, and other ISR genes. We noted that *MESH1* knockdown triggered the induction of *ATF3* mRNAs by RNA-seq (Fig. 1A), a prominent feature of ISR. Therefore, we studied the changes of other ISR markers and noted the upregulation of other ISR genes, including the C/EBP homologous protein (*CHOP*), and cystathionine gamma-lyase (*CTH*) (Fig. 1A). As eIF2α phosphorylation is a feature of ISR, we extracted proteins from RCC4 cells transfected with control or two independent *MESH1* siRNA and determined the status of eIF2α phosphorylation using Western blots (Fig. 2A). *MESH1* siRNAs successfully reduced the MESH1 protein levels (Fig. 2A) and increased the eIF2α phosphorylation (Fig. 2A). Giving that eIF2α phosphorylation reduces the global protein synthesis, this is consistent with the repressed ribosomal pathways at the transcriptome levels in the GSEA analysis (Fig. 1D). eIF2α phosphorylation is often associated with increased *ATF4* translation through the switching between different upstream ORFs [24]. Consistently, *MESH1* knockdown increased the level of ATF4 protein (Fig. 2A), a key transcriptional regulator of the ISR known to activate *ATF3*, *CTH*, and *CHOP*. Therefore, after confirming the successful knockdown of *MESH1* RNA (Fig. 2B), we found *MESH1* knockdown induced the expression of *ATF3* and *CTH* transcripts in RCC4 cells (Fig. 2C, D). Furthermore, we found that *MESH1* knockdown also induced the upregulation of *CHOP* in RCC4 cells (Fig. 2E) and 786-O cells (Supplementary Fig. 1A, B). Together, these data suggest that *MESH1* knockdown activates the PERK branch of ER stress pathway.

Besides PERK pathway, we further examined the potential of *MESH1* knockdown to trigger the ATF6 and IRE1 branches of ER stresses. *MESH1* knockdown induced the expression of *HERPUD1* (Fig. 2F) and *XBP1* total mRNA expression (Fig. 2G), suggesting the activation of ATF6. The activated *IRE1* catalyzes the excision of an intron from *XBP1*-unspliced isoform (*XBP1u*) to become *XBP1*-spliced isoform (*XBP1s*). Therefore, the *XBP1s*/*XBP1u* ratio reflects the IRE1 activity and determines the folding capacity in ER [25]. qRT-PCR revealed that *MESH1* knockdown significantly increased the *XBP1s*/*XBP1u* ratio in RCC4 and 786-O cells (Fig. 2H, Supplementary Fig. 1C) as well as XBP1s target gene *ERdj4* (Fig. 2I). Taken together, these data indicate that *MESH1* knockdown actives all three branches of ER stress pathway.

### The role of ATF4 in the transcriptional response to MESH1 knockdown

**Fig. 3:**
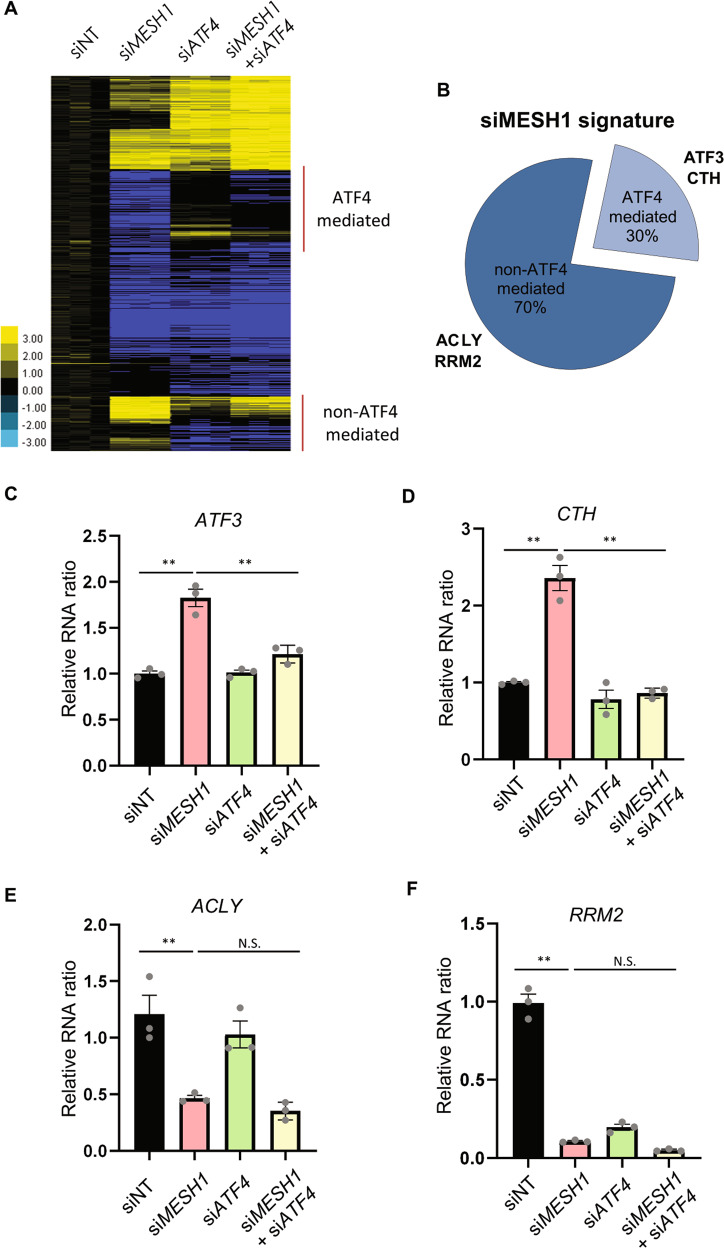
Simultaneous *ATF4* knockdown abolished a portion of *MESH1*-knockdown signature. **A** Heatmap of the transcriptional response of RCC4 to *MESH1* knockdown alone or in combination with *ATF4* knockdown profiled by the array. **B** Transcriptional signature of *MESH1* silencing (si*MESH1* signature) is defined as differential gene expression with fold change > 1.41 with *t* test *P* < 0.05. *ATF4*-mediated genes are defined as having a si*MESH1* signature that is reversed upon simultaneous *ATF4*-silencing with fold change > 1.41 and *t* test *P* < 0.05 compared to *MESH1*-knockdown toward the direction of siNT samples. Note that genes affected by si*ATF4* alone are identified as an off-target effect (due to the low expression level of *ATF4* in unstressed RCC4 cells) and are excluded from the analysis. **C**, **D** Simultaneous *ATF4*-knockdown abolished the *ATF4*-targeted genes *ATF3* (**C**) and *CTH* (**D**) induced by *MESH1*-knockdown. **E**, **F** Simultaneous *ATF4*-knockdown did not alter *ACLY* (**E**) and *RRM2* (**F**) induced by *MESH1* knockdown in RCC4 cells. mRNA abundance was determined by rt-qPCR, normalized by β-actin, and presented in relative ratio to non-targeting control (**C–F**). Statistical analysis: ANOVA with Tukey HSD post-hoc test, **P < 0.01.

*ATF4* is the main transcription factor responsible for the transcriptional feature of ISR [15]. To determine the contribution of *ATF4* to the transcriptome response to *MESH1* knockdown, we compared the transcriptional response of RCC4 to *MESH1* knockdown alone or in combination with *ATF4* knockdown (Fig. 3A, GEO: GSE114128). The heatmap shows that the *MESH1* and *ATF4* double knockdown mitigated a portion of the transcriptional response of *MESH1* knockdown (Fig. 3A). However, a significant portion of the transcriptional response of *MESH1* knockdown was not affected by the *ATF4* knockdown (Fig. 3A). In general, approximately 30% (294 out of 980 genes) of the *MESH1*-knockdown signature was affected by simultaneous *ATF4* knockdown (Fig. 3B), suggesting that *ATF4*-mediated integrated stress response is a notable feature of *MESH1*-knockdown signature. Next, we used qRT-PCR to validate the *ATF4*-mediated vs. non-*ATF4*-mediated transcriptional response to *MESH1* knockdown. We examined the RNA expression of two known target genes of *ATF4*, *ATF3*, and *CTH* (Fig. 3C, D). *MESH1* knockdown upregulated *ATF3* and *CTH*, and these upregulations were indeed abolished by simultaneous *ATF4* knockdown (Fig. 3C, D). We also validated the *MESH1*-knockdown responsive gene that was not affected by simultaneous *ATF4* knockdown (Fig. 3E, F). We found that *ACLY* (ATP citrate synthase) (Fig. 3E) and *RRM2* (Fig. 3F) were both repressed upon MESH1 knockdown, but these changes were not affected by simultaneous *ATF4* knockdown (Fig. 3E, F). Collectively, these data suggest the *ATF4*-mediated transcriptional profile contributed significantly to a portion of the transcriptional response to *MESH1* knockdown. However, *MESH1* knockdown also triggered ATF4-independent transcriptional changes.

### The functional role of *ATF4* and ISR in the *MESH1* knockdown

**Fig. 4:**
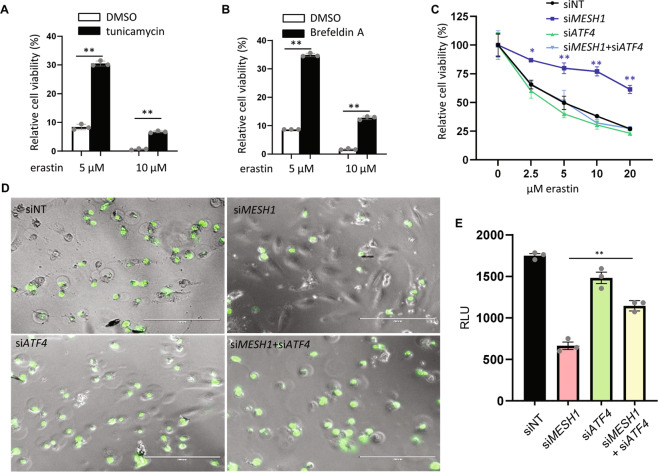
ISR induced by *MESH1* knockdown attenuates ferroptosis. **A**, **B** Tunicamycin and brefeldin A treatment prevents erastin-induced cell death. RCC4 cells were treated with erastin and in combination with tunicamycin (6 µg/ml) (**A**) or brefeldin (2 µg/ml) (**B**). The relative cell viability was determined by CellTiter Glo assay. **C**, **D** *ATF4*-knockdown abolished the ferroptosis survival phenotype of *MESH1*-knockdown in RCC4 cells as determined by CellTiter Glo assay (**C**) or CellTox Green assay to observe the membrane rupture under erastin treatment (5 µM) (**D**) and quantified in (**E**). Statistical analysis: ANOVA with Tukey HSD post hoc test, *P < 0.05, **P < 0.01.

Previously, we found that *MESH1* knockdown protects RCC4 cells from ferroptosis [18]. To investigate the role of ISR in the stringent responses induced by *MESH1* knockdown, we first evaluated whether the activation of ISR can prevent ferroptosis. Given tunicamycin can inhibit the N-linked glycosylation of proteins in ER to trigger ISR [26], we treated cells with erastin alone or together with tunicamycin (Fig. 4A). We found that the treatment of tunicamycin significantly protected cells from ferroptosis, consistent with previous reports [27–29] (Fig. 4A). Brefeldin A can also trigger ISR by targeting protein transport from ER to Golgi [30]. Similar to tunicamycin, brefeldin A also protected cells from erastin-induced ferroptosis (Fig. 4B). These data suggest that ISR triggered by tunicamycin or brefeldin can protect cells from ferroptosis. Given our data suggests that *MESH1* depletion triggers ISR by upregulating ATF4 expression (Figs. 2 and 3), we determined whether the induced *ATF4* may contribute to the ferroptosis protection offered by *MESH1* knockdown. To test this possibility, we determined if simultaneous *ATF4* knockdown would mitigate the ferroptosis protection phenotypes of *MESH1* knockdown. Indeed, the *ATF4* knockdown abolished the ferroptosis survival phenotype of *MESH1*-knockdown cells by the CellTiter Glo assay (Fig. 4C). We further validated the results using the CellTox Green assay to observe the membrane rupture under erastin treatment (Fig. 4D) as quantified in Fig. 4E. In this assay, the dye enters the cells after membrane rupture and then binds DNA to display green fluorescent signals. We found that the protective effect of *MESH1* knockdown under erastin treatment was abolished by simultaneous *ATF4* knockdown. These results indicate that the ferroptosis-resistance phenotype in *MESH1*-knockdown cells may require the activation of the *ATF4* and integrated stress response pathway.

### The potential contribution of enzymatic activities of MESH1 to the cellular responses to *MESH1* knockdown

**Fig. 5:**
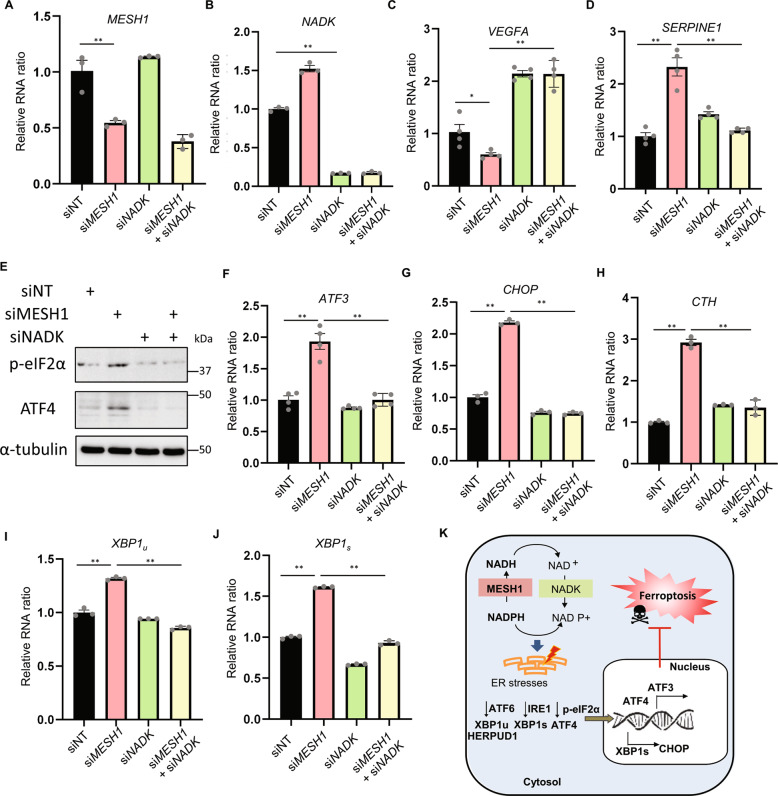
The NADPH phosphatase activity of MESH1 regulates a subset of genes. **A**, **B** RCC4 cells transfected with non-targeting (NT) siRNA, *MESH1*-targeting siRNA, *NADK*-targeting siRNA, or both *MESH1-* and *NADK*-targeting siRNA were validated by its MESH1 (**A**) and NADK levels (**B**). **C**, **D** NADPH phosphatase activity of *MESH1* regulates the mRNA level of *VEGFA* (**C**), and *SERPINE1* (**D**). **E–H** the PERK pathway activated by *MESH1* knockdown was shown to be mitigated by co-depletion of NADK as determined by WB of the eIF2α phosphorylation (**E**), ATF4 protein expression (**E**), and the mRNA levels of *ATF3* (**F**), *CHOP* (**G**), *CTH* (**H**). **I**, **J** ATF6 target gene, total *XBP1* (*XBP1u*) (**I**) and IRE1 target gene, spliced XBP1(*XBP1s*) (**J**) shown to be activated by *MESH1* knockdown were also mitigated by concurrent knockdown of *MESH1* and *NADK*. **K** Schematic illustration ISR triggered by *MESH1* knockdown in preventing ferroptosis. Statistical analysis: ANOVA with Tukey HSD post hoc test, *P < 0.05, **P < 0.01.

We have previously found that MESH1 is an NADPH phosphatase that regulates the level of NADPH during the process of ferroptosis [18]. MESH1 mediates the dephosphorylation and degradation of cytosolic NADPH [18]. Importantly, the cytosolic NADPH can be generated by NAD kinase (NADK) by phosphorylating NADH [31]. Therefore, *MESH1*-depleted phenotypes, such as ferroptosis protection [32], were genetically “suppressed” by the knockdown of *NADK* through the suppression of the cytosolic NADPH level [18]. We thus transfected RCC4 cells with siRNA targeting *MESH1* and *NADK*, either alone or in combination for three days (Fig. 5A, B), and examined the transcriptional changes. First, we validated two genes shown in our RNA-seq (Fig. 1A) to be regulated by the NADPH phosphatase activity of MESH1. Since the MESH1 was induced by extracellular nutrient (cystine) deprivation [18], we focused on genes that encoded proteins that modulate tumor angiogenesis and microenvironments, including vascular endothelial growth factor A (*VEGFA*) and Serpin Family E Member 1 (*SERPINE1*). We found that the downregulated VEGFA upon *MESH1* knockdown was mitigated by concurrent NADK knockdown (Fig. 5C). Also, the induction of SERPINE1 upon *MESH1* knockdown was also mitigated by concurrent NADK knockdown (Fig. 5D). These data suggest that NADPH levels upregulated by *MESH1* knockdown may regulate a subset of the transcriptional response. Therefore, we further examined whether the PERK pathway of ER stress was regulated similarly by measuring p-eIF2α and ATF4 protein levels by Western blots (Fig. 5E) and the mRNA levels of *ATF3* (Fig. 5F), *CHOP* (Fig. 5G), and *CTH* (Fig. 5H). We found that the induction of different molecular features of the PERK pathway by *MESH1* knockdown can be mitigated by the co-depletion of *NADK* (Fig. 5E–H). Similar effects were also observed in ATF6 (XBP1_u_) (Fig. 5I) and IRE1α (XBP1_s_) (Fig. 5J) pathways. Collectively, these data implicate the role of the NADPH phosphatase activity of MESH1 in regulating ER stress. This is consistent with the previous observation that the knockdown of NADK mitigates the ferroptosis protection phenotypes associated with *MESH1* knockdown [18]. Together, while our previous findings showed the crucial role of MESH1 as an NADPH phosphatase in regulating the NADPH level and ferroptosis, these results suggest that NADPH levels regulated by MESH1 may trigger ER stress and ISR as another mechanism to determine ferroptosis (Fig. 5K).

## Discussion

Here, we have shown that the *MESH1* knockdown leads to a reproducible activation of the different branches of ER stresses, including PERK, IRE1, and ATF6. Therefore, MESH1 removal triggers ER stress and represents a form of mammalian stress response pathway. Given the ability of MESH1 to dephosphorylate NADPH, the *MESH1* knockdown has been shown to increase NADPH and protect ferroptosis [18]. Here, we found that NADK knockdown also abolishes the different features of the ER stress activation of *MESH1* knockdown, suggesting a critical role of NADPH accumulation. Our results are consistent with several studies drawing the connections between NADPH and ISR or ER stress. For example, Hexose-6-phosphate dehydrogenase (*H6PD*) is an ER enzyme that generates NADPH and activates different arms of UPR, including *ATF4*, *XBPs*, and *CHOP* induction [33]. Another recent study also showed that elevated NADPH from the TCA cycles triggers the UPR in cancer cells as a metabolic sensing mechanism in ER [34]. Therefore, the accumulation of the NADPH upon *MESH1* knockdown may also contribute to the ISR in the cancer cells. Such finding is also supported by an independent study of RSH in *Caenorhabditis elegans* [35]. However, the detailed mechanisms by which *MESH1* removal and NADPH accumulation lead to ISR remains to be investigated in the future.

It is important to note that the *MESH1* knockdown triggers a transcriptional program highly reminiscent of the transcriptional response of bacterial stringent response [1] and to *Mesh1* removal in Drosophila [16]. Although *MESH1* knockdown leads to activation of the *ATF4* and ISR, these changes only contribute to ~30% of the transcriptional response. Therefore, a significant portion of the transcriptional response to *MESH1* knockdown was independent of ATF4. In addition to eIF2a-ATF4, *MESH1* depletion also activates the *IRE1* and *ATF6*, indicating that *MESH1* knockdown and NADPH accumulation activates all three branches of the ER stress response. Furthermore, the GO and GSEA analysis of the transcriptome responses to *MESH1* knockdown showed the repression of the DNA proliferation, cell cycles, and mitotic pathways with significant similarities with the transcriptome response to the removal of Drosophila *Mesh1* [16] and bacterial stringent response. Therefore, *MESH1* removal may trigger a form of mammalian stringent-like stress response pathway beyond ER stress. An important distinction between the bacterial stringent response and the mammalian counterpart is that bacterial ppGpp is a signaling molecule whose level is dramatically induced during stress and binds to the RNA polymerase to mediate the transcriptome, whereas NADPH serves as an essential agent to mediate the lipid biosynthesis and regeneration of the GSH to overcome oxidative stresses. Together with previous studies [16, 18], these results suggest significant evolutional conservation of phenotypic and transcriptional response to the *MESH1* removal across kingdoms.

*MESH1* knockdown has been shown to confer resistance to ferroptosis induced by oxidative stresses [18]. The activation of ER stress has been shown to confer stress survival to various metabolic stresses [15, 20]. For example, *ATF4* upregulation confers the survival of amino acid deprivation [14], combined hypoxia and lactic acidosis [10, 13], and various tumor therapeutics [36, 37]. Therefore, we expect that *MESH1* knockdown may enable stress survival under stress conditions beyond ferroptosis. While the role of MESH1 beyond ferroptosis remains to be established, its functional relevance and connection with other well-established stress responses will be actively investigated in the future.

## Methods

### Gene-silencing using RNAi

RCC4 cells or 786-O cells were plated in a 6-well plate with 10^5^ cells per well. After 18 h of incubation, the cells were transfected with 40 pmol of siRNA and 3 μL of Lipofectamine RNAiMAX for 72 h. The efficacies of this siRNA were then validated by rt-qPCR or Western blots. Non-targeting siRNA (siNT) (Qiagen, AllStars Negative Control siRNA, SI03650318). Other siRNA includes si*MESH1*#1 (target sequence GGGAAUCACUGACAUUGUG, D-031786-01, Dharmacon), si*MESH1*#2 (target sequence CTGAAGGTCTCCTGCTAACTA, SI04167002, Qiagen), si*ATF4* (target sequence GAUCAUUCCUUUAGUUUAG, CAUGAUCCCUCAGUGCAUA, GUUUAGAGCUGGGCAGUGA, CUAGGUACCGCCAGAAGAA, M-005125-02, Dharmacon), si*NADK* (target sequence UGAAUGAGGUGGUGAUUGA, CGCCAGCGAUGAAAGCUUU, GAAGACGGCGUGCACAAU, CCAAUCAGAUAGACUUCAU, M-006318-01, Dharmacon). If not specified, si*MESH1* indicated si*MESH1*#1.

### Cell culture and reagents

The RCC4 cell line was a gift from Denise Chan (University of California, San Francisco, San Francisco, CA) and was authenticated by DDC (DNA Diagnostics Center) Medical using the short tandem repeat method and tested to be mycoplasma-free in November 2015. 786-O cells were purchased from the Cell Culture Facility at Duke University (Durham, NC, USA). 786-O cell line has been authenticated by STR DNA profiling and tested to be mycoplasma-free by Duke Cell Culture Facility. All cells were cultured in DMEM with 4.5 g/L glucose and 4 mM Glutamine (11995-DMEM, ThermoFisher Scientific) and 10% heat-inactivated fetal bovine serum (Hyclone # SH30070.03HI) in a humidified incubator, at 37 °C with 5% CO_2_. Tunicamycin (T7765) and brefeldin A(B7651) were purchased from Sigma. Erastin was synthesized at Small Molecule Synthesis Facility at Duke University.

### Western blots

Western blotting was performed as previously described [38]. After 72 h of siRNA transfection, RCC4 cells were washed once with ice-cold PBS, then resuspended in RIPA buffer with protease and phosphatase inhibitors. The samples were then lysed by a constant vortex for 30 min at 4 °C, then spun down at 13,000 rpm for 10 min at 4 °C. The protein concentration of the supernatant was determined by the Pierce BCA protein assay kit (#23225, ThermoFisher). Around 40 μg of the protein samples were loaded on 10% sodium dodecyl sulfate-polyacrylamide gel electrophoresis gels, transferred to PDVF membrane, blocked with 5% non-fat milk in TBST. The PVDF membranes were then incubated with primary antibodies overnight at 4 °C. Primary antibodies: MESH1 (1:1000, HPA040895, Sigma); p-eIF2α (1:1000, #9721, Cell signaling); ATF4 (1:500,60035-1, Proteintech); α-tubulin (1:1000, sc-32293, Santa Cruz); β-tubulin (1:1000, #2128, Cell Signaling).

### Quantitative real-time PCR

Total RNA from the samples was extracted using the RNeasy mini kit (Qiagen, 74104) following the manufacturer’s instructions. Total RNA (1.5 µg) was reverse transcribed to cDNA by random hexamers and SuperScript IV (Invitrogen). Power SYBRGreen Mix (ThermoFisher Scientific, 4367659) and StepOnePlus Real-time PCR system (Applied Biosystems) were then used to quantify relative RNA ratio. Samples were biologically triplicated for mean ± SEM. The primer sequences for RT-qPCR are listed in Supplementary Table 3.

### Transcriptome analysis

RNA quality was assessed using an Agilent BioAnalyzer (Agilent). cDNA library was prepared by Illumina TruSeq Stranded mRNA LT Sample Prep Kit – Set A (Illumina, RS-122-2101) according to the manufacturer’s instructions. The library was pooled and sequenced using Illumina HiSeq 4000 with single-end 50 bp read length at The Sequencing and Genomic Technologies Shared Resource of Duke Cancer Institute. The differential analysis was performed using DESeq2 [39]. For cDNA microarray, cDNA was amplified with Ambion MessageAmp Premier RNA Amplification kit (ThermoFisher Scientific, AM1792). The gene expression signatures were interrogated with Affymetrix U133A gene chips and normalized by the RMA (Robust Multi-Array) algorithm. cDNA synthesis and microarray interrogation were performed by the Duke Microarray Core.

### Cell viability and cytotoxicity assays

Cell viability and cytotoxicity assays were performed as previously described [40, 41]. Cell viability of RCC4 cells was determined by CellTiter-Glo luminescent cell viability assay (Promega) following the manufacturer’s protocol. After 48 h of siRNA transfection, RCC4 cells were treated with various doses of erastin. After another 20 h of incubation, CellTiter-Glo substrate (15 µl) was added to the 96-well plate with 100 µl media for 10 min of continuous shaking. The cell viability was then determined by signal intensity using a chemiluminescence plate reader. Cytotoxicity assay was measured by the rupture of the cell membrane using CellTox Green cytotoxicity assay (Promega) by following the manufacturer’s protocol. The fluorescent dye of the CellTox Green assay was added to the media (1:1000). After 30 min of incubation. The cytotoxicity of RCC4 cells as determined by membrane rupture was imaged by fluorescent microscopy and quantified by a fluorescence plate reader.

### Statistical analysis

The number of biological replicates was presented by individual data points in each bar graph. p-values were determined by ANOVA with Tukey HSD post-hoc test in Graphpad. Error bars represent SEM, and significance between samples is denoted as *p < 0.05; **p < 0.01.

### Data deposition

The RNA-Seq and microarray data have been deposited into NCBI GEO with accession numbers: GSE114282 (RNAseq) and GSE114128 (cDNA microarray for si*MESH1* and si*ATF4*).

## Supplementary information

Supplementary information
