# Supplementary material for: The regulation of ferroptosis by MESH1 through the activation of the integrative stress response
Source: Cell Death Dis. 2021 Jul 22;12(8):727. doi: 10.1038/s41419-021-04018-7 (PMC8298397; doi:10.1038/s41419-021-04018-7)
Supplement: Supplementary file 1 — Supplementary information [file 41419_2021_4018_MOESM1_ESM.pdf]

Supplementary Figure 1

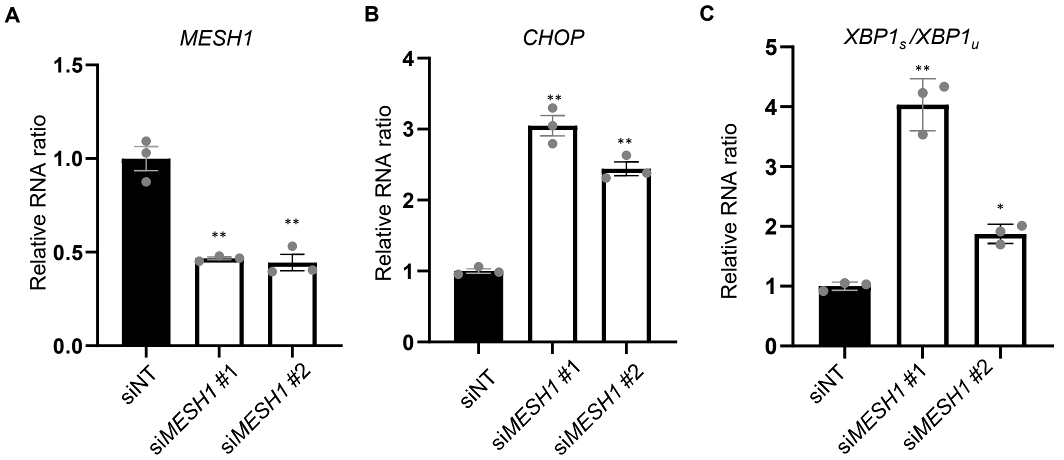

Supplementary Figure 1 *MESH1* knockdown activates the genes involving in ER stress response in 786-O cells.
(**A**) Validation of MESH1 knockdown in 786-O cells. 786-O cells knocked down by non-targeting (NT) siRNA or two independent *MESH1* siRNA for three days were lysed by for qRT-PCR. (**B-C**) *MESH1* knockdown increased RNA expression of genes involving in ER stress response , including *CHOP* (**B**), and *XBP1s*/*XBP1u* ratio (**C**) as determined by qRT-PCR. Statistical analysis: ANOVA with Tukey HSD post-hoc test, **P*<0.05, ***P*<0.01.

Supplementary Table 1 | Top 10 significant Gene Sets of KEGG using Gene Set Enrichment Analysis for *MESH1* silencing cells.

NAME (Depleted Gene Sets)*	p-val	q-val
BASE_EXCISION_REPAIR	0	0.023
DNA_REPLICATION	0.001	0.112
RIG_I_LIKE_RECEPTOR_SIGNALING_PATHWAY	0.005	0.123
CELL_CYCLE	0.001	0.150
BLADDER_CANCER	0.023	0.356
VEGF_SIGNALING_PATHWAY	0.025	0.325
OXIDATIVE_PHOSPHORYLATION	0.012	0.345
HUNTINGTONS_DISEASE	0.008	0.326
FRUCTOSE_AND_MANNOSE_METABOLISM	0.045	0.343
GLYCEROPHOSPHOLIPID_METABOLISM	0.041	0.378

Supplementary Table 2 | Top 10 significant Gene Sets of Gene Ontology using Gene Set Enrichment Analysis for *MESH1* silencing cells.

NAME (Enriched Gene Sets)*	p-val	q-val
CILIUM_MOVEMENT	0	0
CILIUM_MORPHOGENESIS	0	0.087
MOTILE_CILIUM	0	0.060
NEGATIVE_REGULATION_OF_EPITHELIAL_CELL_PROLIFERATION	0	0.069
CILIARY_PLASM	0	0.068
CILIUM_ORGANIZATION	0	0.084
CELL_PROJECTION_ASSEMBLY	0	0.200
MICROTUBULE_BUNDLE_FORMATION	0.009	0.178
SPERM_FLAGELLUM	0	0.161
UBIQUITIN_LIKE_PROTEIN_CONJUGATING_ENZYME_BINDING	0.012	0.153
NAME (Depleted Gene Sets)	p-val	q-val
TRANSLATIONAL_TERMINATION	0	0.021
MITOCHONDRIAL_TRANSLATION	0	0.048
DNA_REPLICATION_INITIATION	0.002	0.362
CELLULAR_PROTEIN_COMPLEX_DISASSEMBLY	0	0.374
HEXOSE_CATABOLIC_PROCESS	0.002	0.531
ORGANELLAR_RIBOSOME	0.001	0.569
NUCLEAR_UBIQUITIN_LIGASE_COMPLEX	0.003	0.498
MONOSACCHARIDE_CATABOLIC_PROCESS	0.003	0.596
INTERMEDIATE_FILAMENT	0	0.538
BASE_EXCISION_REPAIR	0.005	0.501

Supplementary Table 3 | RT-qPCR primers (listed 5' to 3')

MESH1 F'	GAGGCGGGAATCACTGACAT	MESH1 R'	TTGTGCCCCAAAGTGTAGCT
CDK2 F'	GGCCATCAAGCTAGCAGACT	CDK2 R'	CCAGAGTCCGAAAGATCCGG
E2F1 F'	TCGTAGCATTGCAGACCCTG	E2F1 R'	ACATCGATCGGGCCTTGTTT
RRM2 F'	TGGGGACAAAGAGGCTACCT	RRM2 R'	CCAGGCATCAGTCCTCGTTT
ATF3 F'	GTCCATCACAAAAGCCGAGG	ATF3 R'	GGCACTCCGTCTTCTCCTTC
CTH F'	CTCACTGTCCACCACGTTCA	CTH R'	GCCACTGCTTTTTCAAGGCA
CHOP F'	CCTGCAAGAGGTCCTGTCTT	CHOP R'	GCAGGGTCAAGAGTGGTGAA
HerpUD F'	AACGGCATGTTTTGCATCTG	HerpUD R'	GGGGAAGAAAGGTTCCGAAG
uXBP1 F'	TCCGCAGCACTCAGACTACG	u/sXBP1 R'	AGTTGTCCAGAATGCCCAACA
sXBP1 F'	CTGAGTCCGCAGCAGGTG	u/sXBP1 R'	AGTTGTCCAGAATGCCCAACA
Erdj4 F'	GGAAGGAGGAGCGCTAGGTC	Erdj4 R'	ATCCTGCACCCTCCGACTAC
ACLY F'	CTCCGCTCTGCCTATGACAG	ACLY R'	TCCGATGATGGTCACTCCCT
NADK F'	CACAATGGGCTGGGTGAGAA	NADK R'	TTGGACAGGTAGGAGGAGGG
VEGFA_F'	CTTGCCTTGCTGCTCTACCT	VEGFA R'	AGCTGCGCTGATAGACATCC
SERPINE1 F'	GGAGAAACCCAGCAGCAGAT	SERPINE1 R'	TCATCCTTGTTCCATGGCCC
